# Urothelial Cancer Stem Cells

**DOI:** 10.1100/tsw.2010.138

**Published:** 2010-07-20

**Authors:** Irena Dimov, Milan Visnjic, Vladisav Stefanovic

**Affiliations:** University of Nis School of Medicine, Nis, Serbia

**Keywords:** cancer stem cell, urothelium, bladder cancer, tumorigenesis, therapy

## Abstract

There is mounting evidence supporting the idea that tumors, similar to normal adult tissues, arise from a specific stem-like cell population, the cancer stem cells (CSCs), which are considered as the real driving force behind tumor growth, the ability to metastasize, as well as resistance to conventional antitumor therapy. The concept that cancer growth recapitulates normal proliferative and/or regenerative processes, even though in very dysfunctional ways, has tremendous implications for cancer therapy. The rapid development of the CSC field, shoulder to shoulder with powerful genome-wide screening techniques, has provided cause for optimism for the development of more reliable therapies in the future. However, several important issues still lie ahead. Recent identification of a highly tumorigenic stem-like compartment and existence of urothelial differentiation programs in urothelial cell carcinomas (UCCs) raised important questions about UCC initiation and development. This review examines the present knowledge on CSCs in UCCs regarding the similarities between CSCs and the adult urothelial stem cells, potential origin of urothelial CSCs, main regulatory pathways, surface markers expression, and the current state of CSC-targeting therapeutic strategies.

### INTRODUCTION

Within the past 10–15 years, knowledge about cancer and how cancer cells might originate has changed dramatically. There is mounting evidence supporting the idea that tumors, similar to normal adult tissues, arise from a specific stem-like cell population. Accepting the idea that cancer stem cells (CSCs) function like stem cells, but do not always directly arise from or resemble them phenotypically, an American Association for Cancer Research Workshop 2006 defined CSCs as malignant cells with an ability to self-renew and differentiate to form all of the cell types in a given tumor[1].

Adult stem cells are defined as cells that have the ability to perpetuate themselves through self-renewal, but also to generate tissue-specific mature cells through differentiation. In most tissues, these cells are rare. The ability of adult stem cells to be long lived and capable of self-renewal and multilineage differentiation makes them unique and essential in normal physiology[2]. However, like the two faces of Janus, the same qualities might give certain stem-like cells the ability to pose a serious threat to the host organism. CSCs share several characteristics of normal adult stem cells, such as the self-renewal capacity or some differentiation potential. Because most tumors have a clonal origin, the original tumorigenic cancer cell gives rise to phenotypically diverse progeny, including cancer cells with indefinite proliferative potential, the CSC population, as well as cancer cells with limited or no proliferative potential. Therefore, a tumor can be viewed as an “aberrant organ”, initiated and maintained by a relatively small tumorigenic CSC population, which acquired the capacity for indefinite proliferation mostly through accumulated mutations. The concept that cancer growth recapitulates normal proliferative and/or regenerative processes, even though in very dysfunctional ways, has tremendous implications for cancer therapy.

It is generally believed that CSCs, similar to adult stem cells, constitute the minority of the cells within a tumor, but they are nevertheless considered as the real driving force behind tumor growth, self-renewal, and the ability to metastasize[1,2,3,4,5,6]. Delineating normal stem cells and CSC properties, as well as CSC origin, is still the subject of considerable debate. It is generally accepted that CSCs might arise from transformed stem cells or progenitor cells that have regained self-renewal activity; due to the autonomous cell-cycle control, alterations of cellular stress-control mechanisms, and interference of signaling pathways; as a result of accumulated genetic and epigenetic changes[1,2,3,5,6,7,8]. The cell fusion and gene-transfer events have also been linked to several fundamental features of cancer and could be important in the development of the cancer stem cell[9]. In addition to the CSC self-control autonomy that is the hallmark of classical tumor initiation and propagation concept[2,3,7], there is a considerable difference between normal cells and CSCs in their degree of dependence on the stem niche. The CSCs seem to be self-sufficient due to the changes in the cells *per se* and/or changes in the niche signals, where the niche is converted into an environment with dominant signals favoring cell proliferation and growth[5,6].

CSCs have been isolated from cancers of the breast, blood, head and neck, brain, skin (melanoma), thyroid, lung, and organs of the gastrointestinal and reproductive tracts[4]. Current anticancer therapies, such as chemotherapy, radiation, or humanized monoclonal antibodies, mostly fail to eradicate CSC clones and instead may even favor expansion of the CSC pool and/or select resistant self-renewing clones[7,8,9,10]. The rapid development of the CSC field, shoulder to shoulder with powerful genome-wide screening techniques and omics methodology, has already allowed identification of potential CSC molecular targets in many cancers, providing optimism for the development of novel, more reliable, therapies in the future. However, several important issues remain to be resolved.

Urothelial cell carcinoma (UCC) represents a compelling and appropriate cancer that likely involves CSCs. However, the urothelial CSC population has not been isolated indisputably. This review examines the present knowledge on CSCs in UCC regarding the similarities between the CSCs and adult urothelial stem cells, potential origin of urothelial CSCs, main regulatory pathways, surface markers expression, and the current state of CSC-targeting therapeutic strategies.

### EVIDENCE FOR CSCs IN UCC

There is evolving evidence for the existence of CSCs in urologic malignancies. The recent identification of urothelial differentiation programs in UCC further supports the idea that intratumoral heterogeneity in solid epithelial cancers is hierarchical due to the existence and pseudodifferentiation process of the CSC pool[11,12]. Consequently, efforts have been made into translating pathways of CSC initiation and maintenance.

Early clues for the existence of a specific tumor-initiating stem population in UCC arise from the urothelial basal layer[11]. In many stratified polarized epithelia and the urothelium as well, division and differentiation generally proceeds from the epithelial-stromal interface toward the luminal surface, following a hierarchical pattern of growth that is inversely correlated to differentiation potential and replicative activity[13]. The highly differentiated umbrella cells elaborate barrier specialization, protecting basal cells from an assortment of urinary carcinogens. Basal cells constitutively express the 67-kDa laminin receptor (67LR); β4 integrin receptor; high levels of keratins 5, 14, and 17; and low/absent levels of carcinoembryonic antigen-related cell adhesion molecule (CECAM6/CD66), as well as keratins 8 and 18[14,15]. Furthermore, the proliferating cell populations highly express antiapoptotic Bcl-2, Bcl-6, p26, BAG-1, and IAPs[16,17,18], as well as common stem markers, such as CD44 and CD133[19]. The basal layer is the proposed stem/progenitor compartment for the urothelium responsible for the maintenance of tissue homeostasis and renewal. The basement membrane serves as a nidus for epithelial-stromal interactions that are essential for stem cell preservation. In the 3-D culturing condition, *in vitro* cell populations enriched for basal urothelial cells have been proven to give rise to a full-thickness urothelium comprised of intermediate and umbrella cells[12]. BrdU label-retaining cells of rat bladder tissue were exclusively located in the basal layer 1 year after labeling and the proportion of positive basal cells comprised approximately 10%. After cultivation, this cell population showed most of the stem properties[18]. UCC shows a wide degree of heterogeneity within and between tumors. Differentiation properties and the presence of urothelial stem cells may represent a launching point from which to understand cancer cell heterogeneity.

Bladder cancer is the second most common urological malignancy, whereby more than 90% of primary tumors are UCC[20]. Recently, He et al. reported on a highly tumorigenic cell compartment at the edge of tumor nodules that resembles benign urothelial basal cells[21]. Similar to the basal layer, this multipotent compartment resides at the tumor-stroma interface, exchanging signals with the surrounding microenvironment. The cells from this compartment were shown to coexpress the 67LR and the basal cell–specific cytokeratin CK17, and there was a significant enhancement of ErbB and Janus kinase signal pathways. Furthermore, Wnt and Notch pathways, characteristic for stem cells, were highly expressed. In single-cell xenograft suspensions, cells with this phenotype constituted around 13% of the total parental tumor cell population. When injected into animals, they showed similar tumor-forming ability[21].

### ORIGIN OF CSCs IN UCC

The biology of UCC makes it possible to investigate the early stages of tumor development, as recurrences and multiple tumors are common characteristics of this disease. Furthermore, this allows the opportunity to study “reinitiation” of the transforming process in the same genetic and environmental background[22]. Another distinctive characteristic of UCC biology is the dual pathway of carcinogenesis (papillary/noninvasive vs. flat/invasive). The urothelial CSCs seem to play key roles in both[15].

#### Cancer-Related Genetic Profile of Precancerous Lesions and the Normal Urothelium in UCC Patients

Basic cancer research has focused on identifying the genetic changes that lead to cancer. The development of the Genome-Wide Association Study (GWAS) approach has facilitated this goal by unbiased examination of the entire human genome for disease association. This has led to major advances in our understanding of the molecular and biochemical pathways that are involved in tumorigenesis and malignant transformation. The Oncomine database (www.oncomine.com) comprises a plethora of information from the gene expression studies on bladder cancer. There are extensive data that favor the presence of the specific cancer-related genetic profile, as well as epigenetic changes, in precancerous lesions and the morphologically normal urothelium in patients with UCC[23,24,25,26,27]. Detailed histologic-genetic mapping showed that regions with shared genetic aberrations can, in fact, cover a large part of the bladder urothelium[28]. Furthermore, no correlation was found between the genetic chronology and the chronology of the tumor appearance using CGH, LOH, and mutation analyses in patients with recurrent or multiple tumors[29]. A possible scenario could be that a self-renewing cell acquires genetic alteration that partly blocks differentiation or even leads to dedifferentiation (and therefore retains high proliferation potential), followed by field colonization. Most probably, these fields expand over a period of time, up to several years, creating no or only mild symptoms. Islands of altered cells were found to contain additional genetic markers, indicating genomic bias, which may lead to several different preneoplastic and neoplastic lesions[22]. According to these results, it seems that there could be an autonomous, spatially restricted, multiply located, urothelial differentiation program starting from the highly tumorigenic basal compartment, which leads to primary UCC.

### CSC Origin in Noninvasive vs. Invasive UCC

Recently, Brandt et al. proposed that CSCs, for low-grade papillary/noninvasive and high-grade flat/invasive UCC, have a different origin associated with a distinct genetic background. Expression (cDNA) array analyses were employed to define mRNA signatures specifically associated with the two pathways of carcinogenesis. It was hypothesized that papillary/noninvasive carcinomas initially arise from or predominantly differentiate into the intermediate cell phenotype, which has limited replicative potential and does not normally participate in epithelial-stromal interactions that might facilitate invasion. The flat/invasive pathway involves lesions that arise *de novo* or as flat, high-grade carcinoma *in situ* (CIS), and arise from or are at least similar to the basal stem compartment that resides at the tumor-stroma interface, which provides the CSC "pseudo-niche"[15]. Activating mutation in the FGFR3, the most prominent "hit" in papillary carcinomas, occurs in a background of the intact Rb and p53 tumor-suppressor function (the most distinctive markers of invasive carcinoma). Several studies showed no significant evidence for abnormal FGFR3 activation in invasive tumors[20,27]. Furthermore, benign self-limited urothelial papillomas harbor similar mutations in FGFR3, which characterize most of the papillary urothelial carcinomas[27]. As carcinogenesis is a multistep process, accumulation of additional genetic and/or epigenetic alterations in benign lesions might increase their growth potential, resulting in low-grade, noninvasive, papillary tumors.

A gene-profiling study that compared noninvasive to invasive cancers showed that noninvasive urothelial carcinomas predominantly express mRNA-encoding markers of differentiated urothelial cells, including the cell adhesion proteins LAMB3 and ITGB4, and the superficial/umbrella cell marker uroplakin 2[14]. There is strong evidence for basal, intermediate, and superficial differentiation in invasive UCC, but the expression of these differentiation programs in papillary/noninvasive tumors still remains unexamined[15]. Therefore, identification of the origin of CSCs in different forms of UCC still remains an open question and requires a variety of additional studies. However, it should be considered that the variety of prolonged macro- and microenvironmental stimuli, combined with interpersonal inherited genetic and epigenetic stability, may lead to the extent of regulation of pathways involved in transformation, as well as in the reversibility of these changes.

## UROTHELIAL CSC REGULATORY PATHWAYS

Major clinical issues in UCC include the identification of early diagnostics and prediction markers, as well as novel therapeutic targets. Compelling clinical implications of the existence of CSCs have forced numerous studies that relied on empirically selected molecular targets derived from the adult stem cell research. Even though this kind of approach enabled questioning of the validity and stoichiometry of CSC assays, several studies focused on stem cell markers, and the most prominent regulatory molecules of the stem signaling pathways hold some promise for an efficient identification and manipulation of the CSCs[15,30,31].

### Wnt/β-Catenin Pathway

Canonical Wnt signaling is traditionally assumed to play a central role in modulating the delicate balance between stemness and differentiation in several adult stem niches. Wnt/β-catenin signaling is implicated in urothelial development and its proper function in the adult urothelial tissue homeostasis is required[32]. There is a variety of Wnt ligands that bind to the Frizzled receptors and LRP5/6 coreceptors, and several

Wnt-independent pathways resulting in the same intracellular signaling effects. Receptor binding by Wnt ligands rescues β-catenin from a destruction complex that includes the APC tumor-suppressor gene, Axin, and GSK3β[33,34,35]. However, a recent study revealed that Wnt signaling pathway activation could block proper intercellular communication during aging, having a negative effect on stem cell homeostasis and differentiation[36]. Mutations that result in overexpression of Wnt signaling proteins or lead to the disturbance within the Wnt signaling cascade are able to promote tumorigenesis[34]. Furthermore, nuclear β-catenin accumulation appears to provoke epithelial-mesenchymal transformation, promoting tumor-invasive and metastasis potential. In colon cancer, the nuclear β-catenin expression is predominant in tumor cells localized at the invasion front and scattered in the adjacent stromal compartment[34].

Differences in WNT7b expression have been reported between the normal urothelium, superficial, and invasive bladder carcinomas[37]. Several Wnt signaling components are differentially expressed between urothelial CSCs and nontumorigenic cancer cells, including Wnt10a ligand, MYC oncogene, and Cyclin D1[21]. Missense mutations of *APC* were reported in 13% and frameshift deletions in 3% of tumors, all located in regions adjacent to β-catenin binding sites, and it was proposed that the level of β-catenin accumulation correlates with invasiveness[38].

However, mutations typically responsible for deregulated β-catenin accumulation in other tumors, like those in *APC*, *AXIN*, *BTRC*, and *CTNNB1*, are not usual for the urothelial carcinoma[39]. It seems that one of the most prominent mechanisms could be gene silencing of endogenous Wnt inhibitors and Wnt ligand antagonists (such as WIF1, sFRPs, DOC-2/DAB2, DKKs), as a result of DNA promoter methylation[21,39,40,41,42,43]. Methylation and expression levels of six Wnt-antagonist genes (*sFRP-1*, *sFRP-2*, *sFRP-4*, and *sFRP-5*, *Wif-1*, and *Dkk-3*) marked as M score were proposed as a novel epigenetic biomarker panel for UCC. Methylation levels of *sFRP-2* and *Dkk-3* were significant independent predictors of UCC[42]. Expression microarray analyses that have shown the effects of MT1-MMP on cell invasion are mediated in part through changes in DKK3 gene transcription[44].

Further study of the role of the Wnt pathway components and potential cross-talk with other pathways is urgently needed. The identification of key regulatory molecules would offer an opportunity to develop new therapies targeting this pathway in CSCs at different levels[45]. Current knowledge on Wnt antagonists holds some promise to provide novel drugs that target primarily urothelial CSCs[15,45]. Anti-Wnt monoclonal antibodies and even more powerful chimeric molecules (which may also prevent binding to the normal urothelial stem cells), mimicking the effects of Wnt antagonists, may be a fruitful field of future research[44].

Mounting evidence indicates that the activity of the Wnt pathway is influenced by the expression pattern of classic cadherins type I, notably by the status of E-cadherin[46,47,48]. It was shown that the specific E-cadherin genotype could be associated with susceptibility to UCC and loss of E-cadherin expression is associated with a worse clinical prognosis[47]. Besides adhesion, E-cadherin has been shown to act as an inhibitor of β-catenin/TCF-mediated transcription by sequestering β-catenin at the plasma membrane[46,48]. Diminished CDH1 expression that occurs in many variants of UCC, often as a consequence of promoter hypermethylation, may induce inappropriate responsiveness to Wnt factors[32]. In UCC, E-cadherin and p63 have a similar expression variation regarding the tumoral degree of differentiation and the tumoral depth of invasion[49,50,51]. It was previously shown that certain isoforms (TAp63) that are capable of transactivating p53 target genes and inducing cell cycle arrest and apoptosis are expressed in differentiated cells. Other isoforms (ΔNp63) act as dominant-negative factors, inhibiting transcriptional activation of p53 and TAp63, and are mainly associated with stem/reserve-cell populations[52,53]. It was proposed that ΔNp63 isoforms account for p63 protein expression in normal and neoplastic urothelial tissue. Strikingly, in low-grade papillary tumors, p63 expression was predominant in the basal compartment as observed in the normal urothelium, whereas it was frequently lost in high-grade invasive carcinomas. The loss of ΔNp63 correlated with a poor prognosis of invasive UCC[51]. Loss of ΔNp63α followed by N-cadherin up-regulation and ERK signaling activation may be one of the molecular mechanisms underlying progression of invasive UCC[54]. The microarray analysis of 825 UCC samples from 572 patients showed loss of E-cadherin expression accompanied by novel expression of N-cadherin, representing prognostic molecular switching to an invasive phenotype[55].

Analyzing UCC for the expression of E-, P-, and N-cadherin showed P-cadherin retention of the expression in the majority of tumors[56]. P-cadherin–positive staining has been reported to be localized to the basal cell compartment of numerous normal epithelial tissues, including the urothelium, and the restricted distribution of P-cadherin in normal mucosa often overlapped with the proliferative compartment of the tissue[57,58]. P-cadherin staining in low-grade tumors showed the association with an expanded basal cell compartment and in the late-stage invasive lesions, P-cadherin–positive cells were scattered throughout the tumor[56]. Thus, it seems that a specific cadherin expression pattern may have a different role in the urothelial stem cells and urothelial CSC homeostasis.

### Notch Pathway

The Notch pathway is a highly conserved, juxtacrine signaling cascade involved in the development and regulation of differentiation, proliferation, and self-renewal in adult stem cells of various tissues, including different epithelia. Four different Notch receptors (Notch1-4) and five ligands (Jagged-1 and -2 and Delta-like-1, -3, and -4) have been characterized in mammalian cells. Notch signaling may involve a number of other proteins, such as CCN proteins, which are secreted to the extracellular environment where they may act as regulators of the Notch signal. Each Notch monomer is a complex molecule composed of a few functional subunits. The intracellular domain (icN) contains RAM (RBPjk AssociateMolecule) followed by a number of Ankyrin repeats, which mediate the interaction with the Csl (CBF1 in mammals) transcription factor. Additionally, there is a transactivation domain (TAD), two nuclear localization signals (NLS), and a PEST region, which is the site for Notch ubiquitination and therefore functions as a negative regulator. The extracellular face of the Notch transmembrane monomer shows a hydrophobic heterodimerization region, which mediates the binding process. The extracellular Notch monomer is composed of a number of cysteine-rich Lin12 repeats and, in the more distant zone, more than 35 EGF-like repeats that are critical for the interaction with the Notch ligand (fundamentally 11 and 12 EGF-like repeats)[59,60]. Bounding of the ligands induces cleavage of the icN by metalloproteases of the ADAM/TACE family and the γ-secretase enzyme complex comprised of nicastrin, presenilin, and APH-1,PEN-2. Nuclear translocation of icN results in transcriptional activation of the HESs(Hes/E(spl)) and HEYs (Hesr/Hey) gene family through interaction of icN with transcriptional factor Csl and additional proteins (such as Mastermind-like-1, -2, -3). Monoubiquitylation of the ligand by Mindbomb (MIB) induces endocytosis of both ligand and the Notch extracellular domain into the cell where additional signaling might be initiated[59,60,61].

Deregulated expression of wild-type Notch receptors, ligands, and target genes is observed in a growing number of tumors. Among the specific target genes are c-*Myc*, *Skip2*, and *Deltex1*, as well as cell cycle regulators *p27KIP1* and *cyclin* D1[50]. However, the specificity of each ligand for a receptor and the subsequent specificity for downstream target activation are still poorly understood, even in physiological conditions. Depending on the tissue and context, activation of the Notch pathway can lead either to the accumulation of immature progenitor cells or to the terminal differentiation. Thus, the role of this pathway in tumorigenesis must be discussed in the context of the specific tissue[59]. It seems that the result of alteration in Notch signaling is dependent on its normal function in a given tissue[60].

The Notch pathway is one of the key stem signaling pathways that has remained mostly unstudied in UCC. Investigating the role of Notch signaling in UCC development, cross-talk with different growth factors and other stem signaling pathways may provide novel drug targets specific for the CSC population. Notch1-mediated signaling in the epidermis preferentially promotes the commitment of stem cells toward transit populations of cells that are still actively proliferating, but only until they differentiate[62]. Notch1 in primary mouse keratinocytes has shown a tumor-suppressive role[63]. According to the tumor-suppressor role of Notch in the mouse skin, human basal cell carcinomas show reduced Notch1, 2, and Jagged-1 expression[64]. Similarly, the immunohistochemical analysis of the Notch1, 2, and 3, Jagged-1, and Delta-like-1 expression have shown intensive staining in the normal bladder urothelium and a significant decrease in tumor tissues[65].

Expression pattern analysis of the Notch family has shown differences in the papillary/noninvasive UCC vs. flat/invasive UCC. Low-expression of Notch1 and Jagged-1 was proposed as a potential marker for survival of patients with papillary/noninvasive UCC[65].

EGFR signaling overexpression is considered to be one of the key events in the initiation and development of UCC. It is associated with high tumor grade and stage, and could serve as an independent predictor of recurrence and poor prognosis. In the normal urothelium, the regenerative response is driven mostly through the autocrine production of EGR family ligands acting on the EGFR receptor[66,67]. Studies of the response of normal, ‘‘paramalignant’’ cells (genetically modified cells with disabled p53 or p16 functions.) and UCC-derived human urothelial cells to EGF pathway inhibitors have shown that disabled p16 function had no interference in EGFR signal inhibition, whereas loss of p53 function displayed reduced sensitivity to EGFR inhibitors. Malignant cell lines were the most refractory ones, indicating that urothelial cells acquire insensitivity to inhibitors of EGFR signaling pathways as a result of malignant transformation[68]. EGFR activation has been shown to regulate Notch transcription directly in epidermal and cancer cells[69]; therefore, it may be fruitful to analyze the characteristics of cross-talk between these signaling pathways in urothelial CSCs.

Recently, the Notch pathway was identified as a new target in tumor angiogenesis. Notch1 and 4 function in the developing endothelium, whereas Notch3 is critical for smooth muscle cell differentiation. Notch ligands involved in the process of angiogenesis include Delta-like-1, -4, and Jagged-1[70]. Several studies of tumors in mice and humans have shown that Notch components were strongly expressed in tumor vessels, most notably Delta-like-4, compared to adjacent normal vessels[71,72,73]. The expression pattern of Delta-like-4, CD34, and VEGF in UCC compared to normal urothelium indicated that Delta-like-4 is up-regulated in both superficial and invasive UCC. There was significantly strong correlation with CD34 and VEGF expression[74]. The striking pattern of Delta-like-4 expression in tumor vessels prompted several groups to target Delta-like-4/Notch activity. Several preclinical studies have established that VEGF regulates the expression of Notch signaling components. Expression of Delta-like-4 in tumor vessels seems to be directly regulated by high levels of VEGF signaling[75]. Successful blockade of the VEGF pathway has shown reduced tumor growth due to the inadequate tumor angiogenesis. These preclinical results have been validated by successful phase 3 clinical trials of anti-VEGF agents in several types of cancers[70]. The approval of the anti-VEGF monoclonal antibody (bevacizumab) for use in colorectal and lung cancer provides clinical validation for targeting angiogenesis in the cancer therapy. Delta-like-4 antagonists generated a growth inhibition and hypoxia in tumor tissue in a variety of established human and rodent models caused by overgrowth of nonfunctional vessels[75,76,77]. Blockade of Delta-like-4 has shown antitumor effects on some tumors that were resistant to VEGF inhibition[76]. Furthermore, simultaneous blockade of both VEGF and Delta-like-4 resulted in more potent antitumor effects than blockade of either factor alone[78].There is some evidence that other Notch ligands (such as Jagged-1) may also influence tumor angiogenesis[79,80]. Further studies are needed to examine the complex interactions and effects of the Notch pathway in UCC. However, the combinatorial advantage of the VEGF and Delta-Notch pathways may be a potent target for antitumor therapy.

### PTEN/AKT Pathway

The PTEN tumor suppressor is one of the key regulators of cell cycle and stem cell homeostasis in almost all tissues. It is also involved in cell adhesion, migration, and angiogenesis[81]. Mutations/deletions of *PTEN* are one of the most frequent genetic alterations in human cancers. Promoter methylation gene silencing is also found at high frequency in many primary and metastatic human cancers. Germ line *PTEN* mutations have been found in 80% of patients with classic Cowden syndrome, a heritable multiple hamartoma characterized by high frequency of breast, endometrial, and thyroid carcinoma and occasional incidence of other cancers, including bladder and renal cell carcinoma[82].

PTEN is a multifunctional phosphatase that functions as a negative regulator of the phosphatidylinositol 3-kinase (PI3K)/AKT pathway. PI3K activation by growth factors and hormones leads to synthesis of lipid second messenger phosphatidylinositol (3,4,5)-triphosphatase (PIP3). Increased levels of PIP3 promote survival factor AKT activation. AKT is a serine-threonine kinase with three family members (AKT-1, -2, -3) that are ubiquitously expressed, but their levels show divergence highly dependent upon the tissue type. AKT was proven to be involved in a wide array of essential cellular responses regulating several downstream pathways[59,83,84]. Many components of this pathway have been proposed as causal forces in cancer. A number of substrates have been identified, including proapoptotic BAD and pro-caspase-9, as well as transcription factors FOXO3a, TSC2, and GSK-3β, among others. FOXO3a phosphorylation by AKT has been shown to disable its nuclear translocation and up-regulation of target proapoptotic genes and, thus, promote cell survival and proliferation. Activated TSC2 is involved in cellular metabolism and growth regulation due to the mTOR activation[84,85]. AKT is also able to affect cell proliferation and survival by cyclins, cdks, and p21 levels, and their subcellular localization[83].

PTEN dephosphorylates PIP3 leading to AKT and downstream pathways down-regulation. Thus, the resistance to various apoptotic stimuli, disruption in the stem cell self-renewal program, and increased cell growth and cell size could be direct consequences of abnormal AKT activation due to the PTEN deficiency[81,82,84].

However, the predisposition for certain cancer types in Cowden syndrome and sporadic tumor development exhibit strong tissue specificity. Study of PTEN-deficient mice has shown that loss of PTEN function in different tissues elicits drastically different downstream events that may serve as the molecular basis for the differential predilection for tumor formation. The mechanism of tissue specificity in a tumor predisposition as a result of PTEN loss is still unclear[83]. This might be partially due to the various tissue-specific levels of expression of AKT and other proteins, and involvement of signaling pathway bifurcation on multiple levels.

In UCC, a correlation between the decrease of PTEN expression and tumor stage and grade has been shown[86,87,88]. A complex network of associations with other CSC regulators and growth factor signaling pathways slowly emerges. It was proposed that the EGFR pathway activation limits TRAIL-induced apoptosis via an AKT- and XIAP-dependent mechanism in EGFR-dependent human bladder cancer cells[89]. It was recently documented that inhibitors of the PI3K pathway reduced the motility and invasiveness of tumor cells. Furthermore, it was shown that the synergistic effect of *p53* and *Pten* genes deletion was mediated by deregulation of mTOR signaling, consistent with the ability of rapamycin to block bladder tumorigenesis in some preclinical studies[86,87,88,89,90]. However, data considering the role of PTEN and downstream pathways in UCC are sometimes dubious[83,87,91,92,93,94]. In the study of PTEN-deficient mice, given the low apoptosis rate and the full penetrance of the hyperplasia in the urothelium from a very early age, it was striking that the frequency of bladder cancer development showed very low incidence compared with that observed in other tissues[83]. The frequency of PTEN LOH in human bladder cancer was also considerably lower compared to some other tissues[91,92,95,96]. The lack of redundancy of PI3K pathway alterations was also documented, raising the question of single-target therapy efficiency[97].

Many results unquestionably support the hypothesis regarding PTEN functioning as a tumor suppressor in bladder cancer. Alternative AKT-independent mechanisms of PTEN-mediated tumorigenesis must also be considered. It was documented that PTEN directly associates with p53, increasing protein stability, protein levels, and transcriptional activity[98,99]. PTEN may further be involved in cell cycle arrest via interaction with cyclin D and many other targets independently of PI3K. Therefore, positive cooperation between AKT and other PTEN targets is probably necessary in the transforming process[100]. However, it still remains unclear how PTEN dysregulation contributes to the onset or progression of bladder tumors *in vivo* and what role it plays in CSC homeostasis.

### Polycomb Family

The Polycomb family of transcriptional factors silence gene expression (except tritorax proteins), allowing cells to both acquire and maintain differential identity, therefore playing a role in both stem cell self-renewal and CSC homeostasis. The Polycomb group is believed to function by forming multimeric protein complexes, referred to as Polycomb Repressive Complex 1 and 2 (PRC1 and 2), that modify chromatin structure, resulting in target genes repression (such as homeotic selector genes)[100,101,102].

Bmi-1 (a member of PRC1) serves as the gene silencer that induces cellular senescence and cell death, and it can contribute to cancer when improperly expressed. Bmi-1 overexpression (mostly due to the gene amplification) leads to *INK4A*/*ARF* locus repression and consequent inactivation of Rb and p53[103]. In contrast to the many studies on the potential involvement of Bmi-1 in the oncogenesis of various lymphomas and leukemias, there is still a lack of knowledge about its role in the pathogenesis of many solid tumors, including UCC.

Genetic analysis of Bmi-1 expression in UCC showed five-times higher levels compared to the intact tissues. Furthermore, expression of Bmi-1 showed correlation with incidence and progression of bladder tumors[104]. Recent study confirmed overexpression of Bmi-1 protein in UCC that correlated with tumor classification, recurrence, TNM stage, and survival, proposing it as a possible prognostic marker. Notably, Bmi-1 protein was up-regulated to a much greater extent than Bmi-1 mRNA in cancer tissue, suggesting dysregulation at the post-transcriptional level[105]. However, some authors reported no significant Bmi-1 mRNA expression[106]. A genomics approach revealed an 11-gene signature (including *BMI1*) that consistently displayed a stem-cell-resembling expression profile in distant metastatic lesions of different cancers, including bladder cancers[107]. The Bmi-1 overexpression is probably not a primary event in the genetics of UCC, but involved in the progression of the tumor[104].

Other members of the Polycomb family genes also showed strong correlation with disease development, presenting novel potential targets for therapy. Expression levels of CBX7 inversely correlated with the progression of tumor stage and grade in UCC[106]. EZH2 expression showed significant increase in UCC specimens as well as human bladder cancer cell lines[108,109,110]. Strikingly, the Polycomb group proteins are commonly abnormally overexpressed years prior to cancer pathology, making early targeted therapy an option to reverse tumor formation[111].

### UROTHELIAL CSC MARKERS

In the attempt to identify and afterward target tumor-initiating cell populations, the similarities between normal and tumor stem cells of the same tissue have been employed. Many molecules expressed by normal stem cells have been found in their malignant counterparts[112].

The embryonic stem cell marker OCT3/4, the key regulator of self-renewal, showed high expression in human bladder cancer and level of expression significantly correlated with tumor aggressiveness, progression rate, and surveillance of patients[113,114].

CD44 is one of the most prominent stem cell markers. It is a multistructural and multifunctional cell surface molecule involved in cell proliferation; cell differentiation; cell migration; angiogenesis; presentation of cytokines, chemokines, and growth factors to the corresponding receptors; docking of proteases at the cell membrane; as well as in signaling important for cell survival. CD44+ cells are located in the basal layer of the normal urothelium as well as in the UCC[19,115]. A panel of antibodies consisting of cytokeratin 20, p53, and CD44 is almost routinely used for confirmation of bladder cancer[116]. Overexpression of specific CD44 RNA splicing variants, namely CD44v8-10, is significant in various malignant tumors and is considered to be associated with disease progression and metastasis. The ratio in urothelial cancer tissue and urinary exfoliated cells showed a linear and significant correlation in the same patients; therefore, it was proposed as a prognostic predictor. Furthermore, the CD44v8-10- to -standard CD44-ratio (total ratio of all CD44 alternative splicing isoforms) in urothelial cancer was closely associated with tumor progression and aggressiveness[117,118].

Recently, Chan et al.[12] described the detailed isolation and characterization of a tumor-initiating cell subpopulation in primary human bladder cancer, based on the expression of markers similar to that of normal bladder basal cells (lineage CD44+CK5+CK20-). The bladder tumor-initiating subpopulation was further defined functionally by its enriched ability to induce xenograft tumors *in vivo* that recapitulated the heterogeneity of the original tumor. Expression analysis of CD44 in a tissue array of over 300 bladder transitional cell carcinomas revealed that the subpopulation of CD44+ cells comprised around 40% of all tumor cells. The CD44+ subpopulation had 10- to 200-times higher potency to induce tumors with the same phenotype in a xenograft model, confirming their tumor-initiating ability. Strikingly, further examination of eventual heterogeneity within different subsets of CD44+ cancer cells showed a variety of different self-renewal and oncogenic active pathways proteins (80% Gli1, 45% Stat3, 10% Bmi-1, and 5% β-catenin). Interestingly, none of the bladder cancer specimens in this study was found to express either Oct-4 or Nanog in the cytoplasm or nucleus. Despite this molecular heterogeneity, a unique bladder tumor-initiation cell gene signature was identified by gene chip analysis[12]. Another group has described similar CSC populations and investigated the possibility of $EMA^{-}$ $CD44v6^{+}$ as molecular markers of bladder cancer–initiating cells. The $EMA^{-}$ $CD44v6^{+}$ cell population constituted approximately 30% of total cancer cell population. An *in vitro* single-cell cloning assay showed that these cells have a high self-renewal ability and the same clonogenic capacity as the parental tumor[119].

Of particular importance is the fact that CSCs function like stem cells, but do not always resemble them phenotypically. Knowledge on specific CSC markers in UCC may provide a novel method for classification or clinical staging of disease, where the level of CSC presence in the cancer tissue may be connected with a more aggressive cancer phenotype. Furthermore, it may initiate a novel valuable therapeutic approach. Experimental monoclonal antibodies against some surface markers (such as 67LR and CD47) have already given some promising results in human xenografts and studies *in vitro*. CD47 is highly expressed on UCC and functions as a ligand of the SIRP inhibitory molecule expressed on phagocytes. Blockade of CD47 by a monoclonal antibody resulted in efficient and specific macrophage engulfment of bladder cancer cells *in vitro*[12].

### FUTURE DIRECTIONS

As CSC research is yet in its infancy, many promising results have been gained. However, CSC populations have not been indisputably identified and successfully isolated from all forms of UCC. Expression of characteristic differentiation programs in papillary/noninvasive tumors and, therefore, detailed characterization of CSCs and their origin still remains unexamined. UCC of the upper urinary tract has a distinct genetic background and it was shown that urothelial cells lining the urinary tract can be divided into at least three distinct lineages based on the embryonic origin, uroplakin content, keratin expression pattern, and growth potential *in vitro*[120,121]. This all raises the question of CSC characteristics in UCC of the upper urinary tract. There is also a milieu of unusual exogenous carcinogenic and endogenous predisposing factors unique to the upper urothelium, extremely represented in analgesic nephropathy or BEN, making the task to identify and target cancer-initiating cells even more challenging.

CSCs from invasive UCC appear well situated to exchange important signals with adjacent stroma, to escape immune surveillance, and to survive cytotoxic therapy, raising the acute question of specific CSC-targeted therapy[15]. Furthermore, according to present studies, the CSC population may occupy a physical niche at the leading edge of a growing tumor-abutting stroma and vascular structures, which is consistent with the observation that narrow surgical margins are often associated with an increased risk of local recurrence[11,21,22].

Preliminary studies *in vitro* and in mice suggest that blockade of cancer stem cell regulatory pathways may offer a novel and more efficient therapeutic approach, even though there are concerns regarding normal stem cell dysregulation or even partial or total loss of the stem cell pool. Other therapies aimed at CSCs have also shown some promise, but further development will require a more comprehensive understanding of the biology of CSCs and methods for identifying, isolating, and manipulating this cell population. In many cases, different research groups analyzing the same CSC marker or signaling pathway reached contradictory conclusions regarding the correlation between specific molecule expression and disease prognosis, which may be due to the differences in methodology. There is also a need for caution when experimenting with the CSC population and interpreting results of CSC manipulation. Some authors raised the issue of so-called precancerous stem cells that have the ability to differentiate into both benign and malignant lesions under certain conditions[122]. Furthermore, many preneoplastic and neoplastic lesions arise on the chronic inflammation background, where TGF-β, Wnt, and other stem cell signaling cascades are activated to promote the regeneration process[123]. Immune antitumor response *in vivo* raises some elementary questions, even allowing for doubt of relevance of the CSC xenografting assays to evaluate CSC potency to form tumors[15]. Therefore, there is an urgent need to investigate the cross-talk between the immune system and CSCs more thoroughly.

In order to cure UCC, it is necessary and may be even sufficient to eradicate CSCs. The characterization of the molecular features and functional characteristics of the CSC population is still ongoing. The main goal is to delineate a specific set of molecular targets distinct from those expressed on normal urothelial stem cells. If the CSC molecular profile could be established, it might be possible to treat efficiently and in future even prevent UCC.

### ACKNOWLEDGMENTS

This work was supported by a grant, No 145004, from the Ministry of Science and Technological Development of Serbia.
